# Co_3_(PO_4_)_2_·4H_2_O

**DOI:** 10.1107/S1600536808028377

**Published:** 2008-09-13

**Authors:** Young Hoon Lee, Jack K. Clegg, Leonard F. Lindoy, G. Q. Max Lu, Yu-Chul Park, Yang Kim

**Affiliations:** a Department of Chemistry, Kyungpook National University, Daegu 702-701, Republic of Korea; b Centre for Heavy Metals Research, School of Chemistry, F11, University of Sydney, New South Wales 2006, Australia; c ARC Centre of Excellence for Functional Nanomaterials, AIBN, University of Queensland, Brisbane, Queensland 4072, Australia; d Department of Chemistry and Advanced Materials, Kosin University, 149-1 Dongsam-dong, Yeongdo-gu, Busan 606-701, Republic of Korea

## Abstract

Single crystals of Co_3_(PO_4_)_2_·4H_2_O, tricobalt(II) bis­[ortho­phosphate(V)] tetra­hydrate, were obtained under hydro­thermal conditions. The title compound is isotypic with its zinc analogue Zn_3_(PO_4_)_2_·4H_2_O (mineral name hopeite) and contains two independent Co^2+^ cations. One Co^2+^ cation exhibits a slightly distorted tetra­hedral coordination, while the second, located on a mirror plane, has a distorted octa­hedral coordination environment. The tetra­hedrally coordinated Co^2+^ is bonded to four O atoms of four PO_4_^3−^ anions, whereas the six-coordinate Co^2+^ is *cis*-bonded to two phosphate groups and to four O atoms of four water mol­ecules (two of which are located on mirror planes), forming a framework structure. In addition, hydrogen bonds of the type O—H⋯O are present throughout the crystal structure.

## Related literature

Besides crystals of the title compound, crystals of Co_3_(PO_4_)_2_·H_2_O (Lee *et al.*, 2008 ▶) have also been obtained under hydro­thermal conditions. For reviews, synthesis, structures and applications of open framework structures with different cations and/or structure directing mol­ecules, see: Kuzicki *et al.* (2001 ▶); Chen *et al.* (2006 ▶); Jiang & Gao (2007 ▶); Cheetham *et al.* (1999 ▶); Forster *et al.* (2003 ▶); Jiang *et al.* (2001 ▶); Cooper *et al.* (2004 ▶); Choudhury *et al.* (2000 ▶). The structure of the isotypic mineral hopeite was first described by Liebau (1965 ▶).

## Experimental

### 

#### Crystal data

Co_3_(PO_4_)_3_·4H_2_O
*M* *_r_* = 438.79
Orthorhombic, 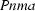

*a* = 10.604 (3) Å
*b* = 18.288 (5) Å
*c* = 5.0070 (13) Å
*V* = 971.0 (5) Å^3^
*Z* = 4
Mo *K*α radiation
μ = 5.46 mm^−1^
*T* = 150 (2) K
0.52 × 0.39 × 0.38 mm

#### Data collection

Siemens SMART 1000 CCD diffractometer
Absorption correction: multi-scan (*SADABS*; Sheldrick, 1999 ▶) *T*_min_ = 0.068, *T*_max_ = 0.125
8780 measured reflections
1228 independent reflections
1166 reflections with *I* > 2σ(*I*)
*R*_int_ = 0.026

#### Refinement

*R*[*F* ^2^ > 2σ(*F* ^2^)] = 0.035
*wR*(*F* ^2^) = 0.102
*S* = 1.16
1228 reflections
101 parameters
10 restraints
H atoms treated by a mixture of independent and constrained refinement
Δρ_max_ = 0.55 e Å^−3^
Δρ_min_ = −1.38 e Å^−3^

### 

Data collection: *SMART* (Siemens, 1995 ▶); cell refinement: *SAINT* (Siemens, 1995 ▶); data reduction: *SAINT* and *XPREP* (Siemens, 1995 ▶); program(s) used to solve structure: *SIR97* (Altomare *et al.*, 1999 ▶); program(s) used to refine structure: *SHELXL97* (Sheldrick, 2008 ▶); molecular graphics: *ORTEP-3* (Farrugia, 1997 ▶), *WebLab ViewerPro* (Molecular Simulations, 2000 ▶) and *POV-RAY* (Cason, 2002 ▶); software used to prepare material for publication: *enCIFer* (Allen *et al.*, 2004 ▶).

## Supplementary Material

Crystal structure: contains datablocks global, I. DOI: 10.1107/S1600536808028377/wm2193sup1.cif

Structure factors: contains datablocks I. DOI: 10.1107/S1600536808028377/wm2193Isup2.hkl

Additional supplementary materials:  crystallographic information; 3D view; checkCIF report

## Figures and Tables

**Table 1:** Selected bond lengths (Å)

Co1—O7^i^	1.901 (3)
Co1—O1	1.918 (3)
Co1—O2^ii^	1.983 (3)
Co1—O2^iii^	1.986 (3)
Co2—O4	2.106 (4)
Co2—O5	2.118 (4)
Co2—O6	2.138 (3)
P1—O7	1.519 (3)
P1—O3	1.521 (3)
P1—O1	1.537 (3)
P1—O2	1.570 (3)

Symmetry codes: (i) 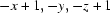; (ii) 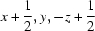; (iii) 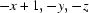.

**Table 2:** Hydrogen-bond geometry (Å, °)

*D*—H⋯*A*	*D*—H	H⋯*A*	*D*⋯*A*	*D*—H⋯*A*
O4—H5⋯O3^iv^	0.894 (10)	2.25 (8)	2.764 (4)	116 (7)
O4—H5⋯O7^v^	0.894 (10)	2.49 (8)	3.209 (3)	138 (9)
O4—H6⋯O3^vi^	0.894 (10)	2.02 (7)	2.764 (4)	140 (10)
O5—H4⋯O3^vii^	0.892 (10)	2.25 (2)	3.133 (5)	170 (8)
O5—H4⋯O3^viii^	0.892 (10)	2.66 (8)	3.133 (5)	115 (6)
O6—H1⋯O1^vii^	0.90 (4)	1.94 (3)	2.690 (4)	140 (4)
O6—H2⋯O7	0.90 (3)	2.35 (3)	3.008 (5)	130 (3)

Symmetry codes: (iv) 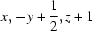; (v) 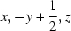; (vi) 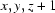; (vii) 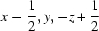; (viii) 
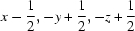
.

**Fig. 1.**
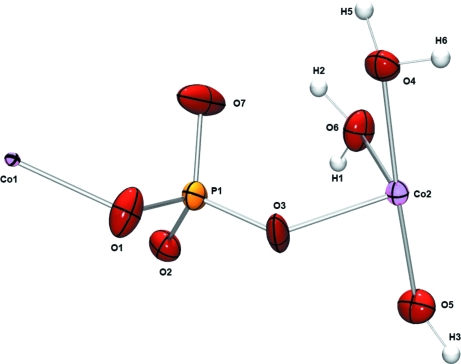
The asymmetric unit of compound (I), drawn with displacement parameters at the 50% probability level. H atoms are given as spheres of arbitrary radius.

**Fig. 2.**
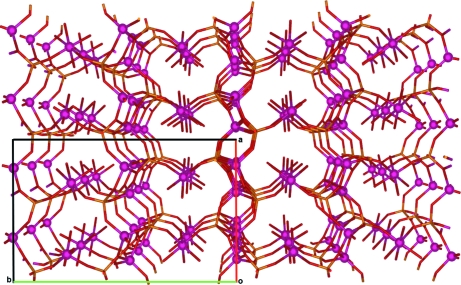
A schematic representation of a section of the three-dimensional network in a projection along [001]. Hydrogen atoms are omitted for clarity.

## supplementary crystallographic information

### Comment

The synthesis and investigation of open-framework transition-metal phosphates has been a growing area of research over recent times. This is not only because of the rich structural chemistry involved, but is also due to many potential applications such as for catalysis, as alternatives for zeolites in separation and storage applications and, in particular, as potential gas storage materials (Kuzicki *et al.*, 2001; Chen *et al.*, 2006; Jiang & Gao, 2007; Cheetham *et al.*, 1999). For example, microporous nickel phosphates incorporating 24-membered rings such as VSB-5 (Versailles/Santa Barbara-5) have been demonstrated to exhibit hydrogen uptake at low temperatures (Forster *et al.*, 2003). Over the past couple of decades, a considerable number of metal phosphates/phosphites with open molecular architectures have also been synthesized incorporating organic units (see, for example: Jiang *et al.*, 2001) and ionic liquids (Cooper *et al.*, 2004) as structure-directing agents, often under hydrothermal or solvothermal conditions. One of the best known families of this type consists of zinc phosphate structures; individual materials of this type can exist as one dimensional (chain and ladder), two dimensional (layer) and three dimensional framework arrangements (Choudhury *et al.*, 2000).

We are currently investigating the synthesis of a variety of similar functional materials through templation effects under hydrothermal conditions. The title compound, Co_3_(PO_4_)_2_.4H_2_O, (I), and the related compound Co_3_(PO_4_)_2_.H_2_O (Lee *et al.*, 2008) were synthesized and structurally characterized as a part of these studies.

The structure of (I) is isotypic with the mineral hopeite, Zn_3_(PO_4_)_2_.4H_2_O (Liebau, 1965) and contains two different Co^2+^ centres bridged by orthophosphate anions (Fig. 1). The coordination environment of Co1 is slightly distorted tetrahedral while that of Co2 is close to octahedral (Table 1). Co1 is bonded to the O atoms of four different phosphate ligands, while Co2 is bonded to the O atoms of two orthophosphate ligands in a *cis*-arrangement. The other coordination sites are occupied by O atoms of the water ligands. A mirror plane passes through Co2 and two of the water molecules (O4 and O5). This coordination geometry leads to the formation of a three-dimensional framework (Fig. 2). A number of hydrogen bonding interactions O—H···O are present and stabilize the structure (Table 2).

### Experimental

H_3_PO_4_ (85%_wt_, 2.3 g, 20 mmol) was added to an aqueous solution (20 ml) of Co(NO_3_)_2_.6H_2_O (2.6 g, 9 mmol) with stirring for 30 min and the ionic liquid, 1-butyl-3-methylimidazolium bromide (2.7 g, 9 mmol), was added dropwise under continuous stirring. The mixture was transferred to a teflon-coated autoclave, heated at 453 K for 3 d and then allowed to cool slowly. A mixture of plate-like and prismatic purple crystals had formed and was filtered off. The crystals were washed with water, dried under vacuum and were manually separated under a microscope. The yields were approximately 0.4 g of the plate-like crystals of the compound Co_3_(PO_4_)_2_.H_2_O (Lee *et al.*, 2008) and and 0.2 g of the prismatic crystals of compound (I).

### Refinement

Water H atoms were located in difference Fourier maps and were refined with *U*_iso_(H) values fixed at 1.5*U*_eq_ of the parent O atoms. O—H bond length restraints of 0.89 (1) Å were also employed. The highest peak and the deepest hole in the final Fourier map are located 0.49 Å from Co1 and 0.33 Å from P2, respectively.

### Crystal data

**Table:**

Co_3_(PO_4_)_3_·4H_2_O	*F*(000) = 860
*M**_r_* = 438.79	*D*_x_ = 3.002 Mg m^−^^3^
Orthorhombic, *P**n**m**a*	Mo *K*α radiation, λ = 0.71073 Å
Hall symbol: -P 2ac 2n	Cell parameters from 5943 reflections
*a* = 10.604 (3) Å	θ = 2.9–28.3°
*b* = 18.288 (5) Å	µ = 5.46 mm^−^^1^
*c* = 5.0070 (13) Å	*T* = 150 K
*V* = 971.0 (5) Å^3^	Prism, purple
*Z* = 4	0.52 × 0.39 × 0.38 mm

### Data collection

**Table:**

Siemens SMART 1000 CCD diffractometer	1228 independent reflections
Radiation source: sealed tube	1166 reflections with *I* > 2σ(*I*)
graphite	*R*_int_ = 0.026
ω scans	θ_max_ = 28.3°, θ_min_ = 2.2°
Absorption correction: multi-scan (SADABS; Sheldrick, 1999)	*h* = −13→13
*T*_min_ = 0.068, *T*_max_ = 0.125	*k* = −24→24
8780 measured reflections	*l* = −6→6

### Refinement

**Table:**

Refinement on *F*^2^	Primary atom site location: structure-invariant direct methods
Least-squares matrix: full	Secondary atom site location: difference Fourier map
*R*[*F*^2^ > 2σ(*F*^2^)] = 0.035	Hydrogen site location: difference Fourier map
*wR*(*F*^2^) = 0.102	H atoms treated by a mixture of independent and constrained refinement
*S* = 1.16	*w* = 1/[σ^2^(*F*_o_^2^) + (0.059*P*)^2^ + 3.8213*P*] where *P* = (*F*_o_^2^ + 2*F*_c_^2^)/3
1228 reflections	(Δ/σ)_max_ < 0.001
101 parameters	Δρ_max_ = 0.55 e Å^−^^3^
10 restraints	Δρ_min_ = −1.38 e Å^−^^3^

### Special details

**Table:**

Experimental. The crystal was coated in Exxon Paratone N hydrocarbon oil and mounted on a thin mohair fibre attached to a copper pin. Upon mounting on the diffractometer, the crystal was quenched to 150(K) under a cold nitrogen gas stream supplied by an Oxford Cryosystems Cryostream and data were collected at this temperature.
Geometry. All esds (except the esd in the dihedral angle between two l.s. planes) are estimated using the full covariance matrix. The cell esds are taken into account individually in the estimation of esds in distances, angles and torsion angles; correlations between esds in cell parameters are only used when they are defined by crystal symmetry. An approximate (isotropic) treatment of cell esds is used for estimating esds involving l.s. planes.
Refinement. Refinement of *F*^2^ against ALL reflections. The weighted *R*-factor *wR* and goodness of fit *S* are based on *F*^2^, conventional *R*-factors *R* are based on *F*, with *F* set to zero for negative *F*^2^. The threshold expression of *F*^2^ > σ(*F*^2^) is used only for calculating *R*-factors(gt) *etc*. and is not relevant to the choice of reflections for refinement. *R*-factors based on *F*^2^ are statistically about twice as large as those based on *F*, and *R*- factors based on ALL data will be even larger.

### Fractional atomic coordinates and isotropic or equivalent isotropic displacement parameters (Å^2^)

**Table:**

	*x*	*y*	*z*	*U*_iso_*/*U*_eq_	Occ. (<1)
Co1	0.64313 (4)	0.00064 (2)	0.20676 (8)	0.00402 (17)	
Co2	0.26113 (6)	0.2500	0.42866 (12)	0.00838 (19)	
P1	0.39745 (9)	0.09462 (5)	0.27639 (18)	0.0123 (2)	
O1	0.5259 (3)	0.07872 (14)	0.1463 (7)	0.0202 (6)	
O2	0.3026 (2)	0.03968 (15)	0.1432 (5)	0.0148 (5)	
O3	0.3601 (3)	0.17305 (16)	0.2139 (6)	0.0201 (7)	
O4	0.3927 (4)	0.2500	0.7439 (7)	0.0155 (8)	
O5	0.1149 (4)	0.2500	0.1406 (9)	0.0192 (8)	
O6	0.1642 (3)	0.16927 (15)	0.6593 (6)	0.0197 (6)	
O7	0.4000 (4)	0.08075 (16)	0.5755 (6)	0.0311 (8)	
H1	0.098 (4)	0.161 (3)	0.553 (10)	0.047*	
H2	0.2194 (15)	0.1390 (19)	0.739 (9)	0.047*	
H3	0.132 (8)	0.280 (5)	0.005 (15)	0.047*	0.50
H4	0.044 (5)	0.231 (5)	0.204 (18)	0.047*	0.50
H5	0.403 (13)	0.296 (3)	0.80 (3)	0.047*	0.50
H6	0.366 (13)	0.210 (4)	0.83 (3)	0.047*	0.50

### Atomic displacement parameters (Å^2^)

**Table:**

	*U*^11^	*U*^22^	*U*^33^	*U*^12^	*U*^13^	*U*^23^
Co1	0.0038 (3)	0.0055 (3)	0.0027 (3)	0.00152 (13)	0.00023 (14)	0.00025 (13)
Co2	0.0081 (3)	0.0088 (3)	0.0082 (3)	0.000	0.0006 (2)	0.000
P1	0.0179 (5)	0.0077 (4)	0.0114 (4)	0.0004 (3)	−0.0004 (3)	0.0003 (3)
O1	0.0128 (13)	0.0123 (12)	0.0357 (16)	−0.0008 (10)	−0.0008 (12)	0.0038 (12)
O2	0.0135 (12)	0.0177 (12)	0.0134 (11)	−0.0024 (10)	0.0020 (10)	−0.0028 (10)
O3	0.0331 (17)	0.0099 (13)	0.0174 (14)	0.0061 (11)	0.0094 (11)	0.0022 (10)
O4	0.017 (2)	0.0169 (19)	0.0130 (16)	0.000	−0.0012 (15)	0.000
O5	0.0136 (18)	0.026 (2)	0.0183 (18)	0.000	−0.0003 (16)	0.000
O6	0.0175 (14)	0.0149 (13)	0.0267 (14)	−0.0024 (11)	0.0027 (12)	0.0026 (12)
O7	0.066 (2)	0.0131 (13)	0.0142 (13)	−0.0105 (14)	−0.0095 (15)	0.0020 (10)

### Geometric parameters (Å, °)

**Table:**

Co1—O7^i^	1.901 (3)	P1—O7	1.519 (3)
Co1—O1	1.918 (3)	P1—O3	1.521 (3)
Co1—O2^ii^	1.983 (3)	P1—O1	1.537 (3)
Co1—O2^iii^	1.986 (3)	P1—O2	1.570 (3)
Co2—O3^iv^	2.058 (3)	O4—H5	0.893 (10)
Co2—O3	2.058 (3)	O4—H6	0.893 (10)
Co2—O4	2.106 (4)	O5—H3	0.892 (10)
Co2—O5	2.118 (4)	O5—H4	0.891 (10)
Co2—O6	2.138 (3)	O6—H1	0.89 (4)
Co2—O6^iv^	2.138 (3)	O6—H2	0.90 (3)
			
O7^i^—Co1—O1	121.18 (15)	O7—P1—O3	111.40 (17)
O7^i^—Co1—O2^ii^	105.64 (13)	O7—P1—O1	111.8 (2)
O1—Co1—O2^ii^	110.16 (12)	O3—P1—O1	108.79 (16)
O7^i^—Co1—O2^iii^	106.57 (12)	O7—P1—O2	108.84 (17)
O1—Co1—O2^iii^	108.97 (13)	O3—P1—O2	110.43 (17)
O2^ii^—Co1—O2^iii^	102.75 (8)	O1—P1—O2	105.46 (16)
O3^iv^—Co2—O3	86.26 (16)	P1—O1—Co1	130.42 (18)
O3^iv^—Co2—O4	93.09 (12)	P1—O2—Co1^v^	128.01 (16)
O3—Co2—O4	93.09 (12)	P1—O2—Co1^iii^	115.24 (15)
O3^iv^—Co2—O5	91.00 (12)	Co1^v^—O2—Co1^iii^	116.58 (13)
O3—Co2—O5	91.00 (12)	P1—O3—Co2	132.08 (17)
O4—Co2—O5	174.38 (15)	Co2—O4—H5	108 (10)
O3^iv^—Co2—O6	178.01 (12)	Co2—O4—H6	98 (10)
O3—Co2—O6	93.16 (12)	H5—O4—H6	133 (3)
O4—Co2—O6	85.03 (11)	Co2—O5—H3	112 (6)
O5—Co2—O6	90.91 (12)	Co2—O5—H4	112 (6)
O3^iv^—Co2—O6^iv^	93.16 (12)	H3—O5—H4	134 (3)
O3—Co2—O6^iv^	178.01 (12)	Co2—O6—H1	100 (4)
O4—Co2—O6^iv^	85.03 (11)	Co2—O6—H2	110.7 (10)
O5—Co2—O6^iv^	90.91 (12)	H1—O6—H2	132 (2)
O6—Co2—O6^iv^	87.36 (16)	P1—O7—Co1^i^	133.79 (19)
			
O7—P1—O1—Co1	41.2 (3)	O1—P1—O2—Co1^iii^	−20.9 (2)
O3—P1—O1—Co1	164.7 (2)	O7—P1—O3—Co2	−22.9 (3)
O2—P1—O1—Co1	−76.9 (3)	O1—P1—O3—Co2	−146.5 (2)
O7^i^—Co1—O1—P1	8.8 (3)	O2—P1—O3—Co2	98.2 (3)
O2^ii^—Co1—O1—P1	−115.1 (2)	O3^iv^—Co2—O3—P1	153.57 (18)
O2^iii^—Co1—O1—P1	132.9 (2)	O4—Co2—O3—P1	60.7 (3)
O7—P1—O2—Co1^v^	34.1 (3)	O5—Co2—O3—P1	−115.5 (3)
O3—P1—O2—Co1^v^	−88.5 (2)	O6—Co2—O3—P1	−24.5 (3)
O1—P1—O2—Co1^v^	154.17 (19)	O3—P1—O7—Co1^i^	143.7 (3)
O7—P1—O2—Co1^iii^	−140.96 (19)	O1—P1—O7—Co1^i^	−94.4 (4)
O3—P1—O2—Co1^iii^	96.45 (18)	O2—P1—O7—Co1^i^	21.7 (4)

Symmetry codes: (i) −*x*+1, −*y*, −*z*+1; (ii) *x*+1/2, *y*, −*z*+1/2; (iii) −*x*+1, −*y*, −*z*; (iv) *x*, −*y*+1/2, *z*; (v) *x*−1/2, *y*, −*z*+1/2.

### Hydrogen-bond geometry (Å, °)

**Table:**

*D*—H···*A*	*D*—H	H···*A*	*D*···*A*	*D*—H···*A*
O4—H5···O3^vi^	0.89 (1)	2.25 (8)	2.764 (4)	116 (7)
O4—H5···O7^iv^	0.89 (1)	2.49 (8)	3.209 (3)	138 (9)
O4—H6···O3^vii^	0.89 (1)	2.02 (7)	2.764 (4)	140 (10)
O5—H4···O3^v^	0.89 (1)	2.25 (2)	3.133 (5)	170 (8)
O5—H4···O3^viii^	0.89 (1)	2.66 (8)	3.133 (5)	115 (6)
O6—H1···O1^v^	0.90 (4)	1.94 (3)	2.690 (4)	140 (4)
O6—H2···O7	0.90 (3)	2.35 (3)	3.008 (5)	130 (3)

Symmetry codes: (vi) *x*, −*y*+1/2, *z*+1; (iv) *x*, −*y*+1/2, *z*; (vii) *x*, *y*, *z*+1; (v) *x*−1/2, *y*, −*z*+1/2; (viii) *x*−1/2, −*y*+1/2, −*z*+1/2.
